# Detection and identification of potentially infectious gastrointestinal and respiratory viruses at workplaces of wastewater treatment plants with viability qPCR/RT-qPCR

**DOI:** 10.1038/s41598-022-08452-1

**Published:** 2022-03-16

**Authors:** Agata Stobnicka-Kupiec, Małgorzata Gołofit-Szymczak, Marcin Cyprowski, Rafał L. Górny

**Affiliations:** Central Institute for Labour Protection – National Research Institute, Czerniakowska 16 Street, Warsaw, Poland

**Keywords:** Biological techniques, Microbiology, Environmental sciences, Natural hazards, Risk factors

## Abstract

This study aimed to qualitatively and quantitatively assess the prevalence of the most common respiratory and gastrointestinal viruses in the air, surface swab, and influent/effluent samples collected in wastewater treatment plants (WWTPs). Application of qPCR/RT-qPCR (quantitative polymerase chain reaction/reverse-transcription quantitative polymerase chain reaction) assays combined with PMA (propidium monoazide) dye pretreatment allowed detecting the potentially infectious and disintegrated viral particles in collected samples. In the air at workplaces in WWTPs, the most frequent isolation with the highest concentrations (reaching up to 10^3^ gc/m^3^ of potentially infectious intact viral particles) were observed in case of adenoviruses (AdVs) and rotaviruses (RoVs), followed by noroviruses (NoVs). Viruses were significantly more often detected in the air samples collected with Coriolis μ impinger, than with MAS-100NT impactor. The temperature negatively (Spearman correlation: –1 < R < 0; *p* < 0.05), while RH (relative humidity) positively (0 < R < 1; *p* < 0.05) affected airborne concentrations of potentially infectious viral particles. In turn, the predominant viruses on studied surfaces were RoVs and noroviruses GII (NoV GII) with concentrations of potentially infectious virions up to 10^4^ gc/100 cm^2^. In the cases of SARS-CoV-2 and presumptive SARS-CoV-2 or other coronaviruses, their concentrations reached up to 10^3^ gc/100 cm^2^. The contamination level of steel surfaces in WWTPs was similar to this on plastic ones. This study revealed that the qualitative and quantitative characteristics of respiratory and gastrointestinal viruses at workplaces in WWTPs is important for proper exposure assessment and needs to be included in risk management in occupational environment with high abundance of microbial pollutants derived from wastewater.

## Introduction

Wastewater is a mixture of domestic, industrial, hospital sewage, and rainwater. It was confirmed to be an important route of transmission for several viral pathogens present in the human population^1^. Viruses are a major causative agent of many diseases like gastroenteritis, hepatitis, and respiratory illnesses, including infections with a lethal course^2^. It is estimated that around 80% of worldwide diseases are waterborne^3^. People suffering from viral infections can excrete 10^5^–10^13^ viral particles per gram of stool. These viruses can persist in the environment for long periods, resulting in their high levels in fecal effluents^4–6^. The degree of wastewater contamination depends on the prevalence of viral infections and characteristics of viruses circulating in a given population^7^. As a result, workers in wastewater treatment plants (WWTPs) may be exposed to viral agents during their occupational activities and, compared to general population, are more likely to develop a wide variety of work-related symptoms, including respiratory and gastrointestinal adverse outcomes^1,8,9^.

Nowadays, noroviruses (NoVs), rotaviruses (RoVs), human bocavirus (HBoV), and adenoviruses (AdVs) are classified as an emerging waterborne viruses, while avian influenza A virus (IAV) and coronaviruses (CoVs) are considered to be potentially emerging waterborne pathogens^7,10^. In the world scale, RoVs (dsRNA; non**-**enveloped; 60–80 nm; the *Reoviridae* family) and NoVs (ssRNA; non-enveloped; 27 nm; the *Caliciviridae* family) are the most common cause of gastrointestinal disorders, with nausea, vomiting, and diarrhea^5,11–14^. In turn, NoVs are associated with gastroenteritis in all age groups, while RoVs are predominant among children^16,17^. Duration of RoVs and NoVs fecal shedding may reach up to 4 weeks and 1 week, respectively^18^.

AdVs (dsDNA; non-enveloped; 70–100 nm; the *Adenoviridae* family) have been reported to be the second most important viral pathogens of gastroenteritis after rotaviruses; however, depending on the species, they can be responsible for different infections, including respiratory and ocular ones as well as meningitis, encephalitis, and hepatitis^4,19–22^.

HBoV (ssDNA; non-enveloped; 18–25 nm; the *Parvoviridae* family) has a global distribution and is associated with respiratory and enteric infections^23^. Despite the detection of viral nucleic acid in stool samples from symptomatic patients, the role of this virus in gastroenteritis remains unclear. The human bocavirus is often present in specimens from healthy individuals and is frequently found in fecal samples containing other well-defined viral pathogens such as HAdVs, NoVs, and RoVs^24^.

IAV (ssRNA; enveloped; 80–120 nm; the *Orthomyxoviridae* family) cause the majority of viral lower respiratory tract infections and may be excreted with stool by infected individuals with very long-term shedding (up to 2 months in case of immunocompromised patients)^25^. Abundance of IAV in wastewater may be increased by surface water sediments present in influent, which act as a long-term source of influenza viruses in the aquatic habitat^26^.

CoVs (ssRNA; enveloped; 60–140 nm; the *Coronaviridae* family) are responsible for human and animal respiratory and gastrointestinal infections. CoVs were considered minor human pathogens because they were usually accountable for common cold or mild respiratory infections in immunocompromised people^27^. Nevertheless, the emergence of a new and highly pathogenic zoonotic disease recently caused by SARS-CoV-2 sheds light on questions that need to be answered in order to target public health responses. CoVs are mainly transmitted through respiratory droplets^28^. However, SARS-CoV-2 RNA has been detected in stool and urine samples of patients showing symptoms of COVID-19 and of asymptomatic carriers as well^29–32^.

Wastewater treatment generates aerosols of different sizes and all airborne biological agents can be subsequently deposited on surfaces^33^. The WWTP workers can be exposed to viral particles either via inhalation of bioaerosol emitted during technological processes (especially in the places where hermetization of treatment tasks is limited) or deglutition after direct contact with contaminated surfaces, clothes or tools.

Nowadays, molecular methods such as polymerase chain reaction (PCR) and quantitative PCR (qPCR) are a ‘gold standard’ in virus detection and identification. Nevertheless PCR-based methods are not able to discriminate between capsid integrated, potentially infectious and damaged, non-infectious viral particles^34^. Propidium monoazide (PMA) is DNA/RNA intercalating dye with a photo-inducible azide group that covalently cross-links to nucleic acids upon exposure to bright light^35^. PMA only crosses damaged membrane barriers, thus coupling PMA with qPCR or RT-qPCR, also called as viability-PCR (v-qPCR/v-RT-qPCR) is promising solution to distinguish potentially infectious and non-infectious viral particles^34^.

There are several studies examining the presence of viruses in wastewater and WWTPs; however, the knowledge about potential infectivity of viruses in this occupational environment is still scarce^1,36,37^. This study is the first investigation qualitatively and quantitatively analyzing presence of the most common gastrointestinal and respiratory viruses in the occupational environment of WWTPs. The aim of this research was to assess the prevalence of these viruses in the air, surface swab and influent/effluent wastewater samples collected in WWTPs with qPCR/RT-qPCR assays combined with PMA dye pretreatment to discriminate potentially infectious and disintegrated viral particles in collected samples.

## Methodology

### Sampling sites and sample types

Bioaerosol and surface swab sampling was performed at workplaces in five different wastewater treatment plants (A, B, C, D, and E – see Table 1). All investigated plants had a minimum capacity of 60,000 m^3^ of wastewater per day and treated municipal and hospital sewage using mechanical–biological technology. All tested workplaces were located indoors and at all of them the treatment processes were open or only partially hermetically sealed. Additionally in this study, the influent and effluent samples were also microbiologically examined. All samples were collected in triplicates in March 2021 (twice in the interval of two weeks) and taken during normal operating hours after obtaining the appropriate permits from the authorities of investigated WWTPs.

**Table 1:** Description of studied wastewater treatment plant (WWTP) sites and number and characteristics of surface swab samples.

WWTP	Site	Performed tasks
A, D	Wastewater pumping section	Pumping wastewater into treatment system
A, B, C, D, E	Screens section	Removal of big objects, screens storage
B, C	Grit chamber section	Removal of heavier solid particles with aeration, grease traps
B, C, D, E	Dewatering/thickening sludge section	Dewatering and thickening of sludge intended for incineration

Workplace	Number of surface swab samples	
	ST	P
Wastewater pumping section	8	6
Screens section	10	7
Grit chamber section	6	4
Dewatering/thickening sludge section	8	6
Total	31	23

*ST* steel, *P* plastic.

### Bioaerosol sampling

In this study, two different instruments were used to collect air samples: Coriolis μ impinger (Bertin Technologies, St-Quentin-en-Yvelines, France) and MAS-100NT impactor (MBV AG, Stäfa, Switzerland)^38,39^. During the measurements, both samplers were placed at a height of 1–1.5 m above the floor level to simulate aspiration from the human breathing zone^40^ and at least 1 m apart to avoid any interference between them. In total, 52 bioaerosol samples (26 Coriolis μ and 26 MAS-100NT, respectively) were collected at the following sampling sites: wastewater pumping Sect. (4 and 4), screens section (10 and 10), grit chamber (4 and 4), and dewatering and thickening sludge section (8 and 8) (Table 1).

A cyclone-based Coriolis μ impinger samples airborne particles into a liquid medium. Each time, the air samples were collected for 10 min at a flow rate of 200 L/min using sterile sampling cones filled with 15 mL of universal viral transport medium (VTM) (Capricorn Scientific GmbH, Ebsdorfergrund, Germany)^41,42^. After each sampling session, the external and internal surfaces of both the impinger inlet and aspiration tube were cleaned and disinfected with isopropyl alcohol, the cone removed from the sampler and the sample stored in 0–4 °C until further analysis.

A single-stage MAS-100NT impactor operates by aspirating the air through a 400-hole perforated inlet plate onto a Petri dish containing biological collection media. Each time, the air samples were collected for 20 min at a flow rate of 100 L/min on standard Petri dishes filled with the bi-phase medium consisting of solid phase mycoplasma base agar (MBA, Oxoid Ltd., Basingstoke, UK) covered with thin layer liquid-phase VTM to maximize the potential of viral particle recovery^39,43^. After each sampling session, an impactor inlet was cleaned and disinfected with isopropyl alcohol. After collection, the samples were transported to laboratory within 12 h where they were stored in − 80 °C until further analysis^44^.

### Surface swab sampling

In total, 54 swab samples were collected from stainless steel and plastic surfaces (machine valves, machine handles, hatch handles, machine controllers, handrails) with sterile polyester fiber-tipped swabs (Deltaswab PurFlock Ultra ViCUM, Deltalab, Barcelona, Spain) prewetted in 0.9% saline solution, which ensures the most effective recovery of viruses from nonporous fomites^45,46^ (Table 1).

### Wastewater samples

Fifteen wastewater influent and the same number of effluent samples were collected (each of them into a sterile 1000 mL glass container) and kept in 4 °C for less than 24 h until further analysis.

### Laboratory analysis

#### Aerosol, surface swab and wastewater samples

All liquid media with air samples were concentrated by ultrafiltration using Amicon Ultra-15 (molecular weight cut-off 30 kDa) centrifugal filter device (Merck Millipore Ltd., Livingston, UK) at 3200 × g for 20 min in 4 °C^41,47^. Centrifugal concentration step was repeated until the entire volume of the sample passed through the filter. The concentrated samples (400 µL) were intended for further analysis. In turn, the swab shafts of swab samples were cut off, then placed into 400 µL of 1 × PBS (pH = 7.2) and vortexed thoroughly using a programmable rotator-mixer (Multi RS-60, Biosan, Riga, Latvia) at 800 rpm for 15 min. Influent and effluent wastewater samples were centrifuged at 4500 × g for 5 min in 4 °C and each obtained supernatant was concentrated as described above^47^.

#### PMA dye pretreatment

All processed samples were divided into two equal aliquots (200 µL). The first one was intended for direct viral DNA/RNA isolation, the second one for PMA dye pretreatment allowing detection of potentially infectious viral particles. In this case, the samples were treated with PMAxx Dye (20 mM in H_2_O; Biotium, Inc., Hayward, USA) for a final concentration of 60 µM^48^. Tubes were gently mixed by inverting several times and then incubated in the dark for 15 min at room temperature with rotation at 200 rpm. The treated samples were exposed to 40 W LED light with a wavelength of 460 nm for 15 min using a photo-activation system (PMA-Lite LED Photolysis Device; Biotum Inc.).

#### Viral DNA/RNA extraction

The extraction of viral DNA/RNA from all samples was carried out with Kogene Power Prep Viral DNA/RNA Extraction Kit CE-IVD (Kogene Biotech, South Korea) according to the manufacturer’s instructions to produce a final volume of 45 μL. Obtained RNA/DNA samples were stored in − 20 °C until further analysis.

#### Quantitative PCR/reverse-transcription quantitative PCR (qPCR/RT-qPCR) and viability quantitative PCR/viability reverse-transcription quantitative PCR (v-qPCR/v-RT-qPCR) assays

Both qPCR/v-qPCR (for DNA viruses) and RT-qPCR/v-RT-qPCR (for RNA viruses) were performed using CFX96 real-time PCR thermocycler (Bio-Rad, Hercules, USA). The detection of AdVs, HBoV, RoVs, NoVs, IAV, and SARS-CoV-2 were carried out with Adenovirus, Bocavirus, Rotavirus, Norovirus (GI and GII), Influenza A, and SARS-CoV-2 VIASURE Real Time PCR Detection Kits (all: CerTest Biotec S.L., Zaragoza, Spain), respectively, according to procedures recommended by the manufacturer. The applied PCR kits have a detection limit of ≥ 10 RNA/DNA copies per reaction.

The target genes employed for PCR-based detection and identification of viruses represent conserved regions with the hexon gene for AdVs, the NSP3 gene for RoVs, the ORF1-ORF2 junction for NoV genogroup I (GI) and NoV genogroup II (GII), the M1 gene for IAV, the ORF1ab and N genes for SARS-CoV-2.

The cycling conditions for DNA viruses were as follows: polymerase activation at 95 °C for 2 min, then 45 cycles of denaturation at 95 °C for 10 s, and annealing at 60 °C for 50 s. In case of RNA viruses, the reverse transcription at 45 °C for 15 min was followed by initial denaturation at 95 °C for 2 min, then 45 cycles of denaturation at 95 °C for 10 s, and annealing at 60 °C for 50 s. According to the manufacturer’s procedure, the fluorogenic data were collected through the FAM, ROX, and HEX channels. Both negative and positive controls, purchased from CerTest Biotec, were included in each run. All samples were tested in duplicates.

All qPCR/RT-qPCR and v-qPCR/v-RT-qPCR data were collected and quantification cycles (Cq) were calculated using CFX96 manager software (Bio-Rad). According to the manufacturer’s instruction, the samples with Cq ≤ 40 for AdVs, HBoV, NoV GI, NoV GII, RoVs, and IAV as well as with Cq ≤ 38 for SARS-CoV-2 were considered as positive. In case of SARS-CoV-2, if only N gene target was positive, the interpretation was presumably positive for SARS-CoV-2 and the differentiation of SARS-CoV-2 from other coronaviruses, including animal ones, requires further analysis. The negative samples and the samples with Cq > 40 were reanalyzed after tenfold dilution to evaluate the possible presence of inhibitors. Quantification analyses were performed based on standard curves, obtained by amplification of positive control tenfold dilutions (standard from 1 × 10^1^ to 1 × 10^7^ gene copies/reaction), and log RNA/DNA copies were plotted against Cq value. All standard curves had efficiencies between 90 and 110% and r^2^ above 0.98.

To minimize the potential contamination, all analytical steps were performed in separate rooms, including RNA/DNA isolation, preparation of reagents, sample preparation, and amplification. All analyzes were carried out using the sterile RNase/DNase-free filter pipette tips only. The obtained results were expressed as the number of viral genome copies per 1 m^3^ of the air (gc/m^3^), per 100 cm^2^ of tested surfaces (gc/100 cm^2^), and per 1 L of influent and effluent wastewater (gc/L).

#### Temperature and relative humidity

During sampling, the temperature and relative humidity of the air were measured using portable thermo-hygrometer (Omniport 20; E + E Elektronik GmbH, Engerwitzdorf, Austria).

#### Statistical analysis

The obtained results were statistically analyzed with Shapiro–Wilk, Fisher Exact, Kruskal–Wallis and Mann–Whitney test as well as Spearman’s rank correlation coefficient using STATISTICA data analysis software system, version 7.1 (StatSoft Inc., Tulsa, USA). Probability values at *p* below 0.05 were considered statistically significant.

## Results

### Presence of viruses in the air, surface, and wastewater samples

The performed qPCR/RT-qPCR-based studies revealed the presence of gastrointestinal and respiratory viral nucleic acids in the air, on surface as well as in influent and effluent wastewater samples. In general, the most commonly detected nucleic acids indicated presence of AdV, RoV, and NoV GII. The most prevalent in the air were AdVs, on surfaces RoVs and NoV GII, in influent samples AdVs and NoV GII, and in effluent samples NoV GII (Table 2).

**Table 2:** Number and percentage of virus-positive and potentially infectious virus-positive air, surface, influent and effluent wastewater samples as identified by qPCR/RT-qPCR in total studied samples and as identified by v-qPCR/v-RT-qPCR among all positive samples.

Viruses		Number and percentage (%) of positive samples				
		Air ^C^*^)^	Air ^M^**^)^	Surface swabs	Influent wastewater	Effluent wastewater
qPCR/RT-qPCR	AdVs	12/26 (46.2)	8/26 (30.8)	23/54 (42.6)	15/15 (100)	6/15 (40)
	HBoV	2/26 (7.7)	ND	16/54 (29.6)	9/15 (60)	3/15 (20)
	NoV GI	4/26 (15.4)	ND	17/54 (31.5)	12/15 (80)	4/15 (26.7)
	NoV GII	6/26 (23.1)	ND	30/54 (55.6)	15/15 (100)	7/15 (46.7)
	RoVs	9/26 (34.6)	5/26 (19.2)	34/54 (63)	11/15 (73.3)	5/15 (33.3)
	IAV	ND	ND	ND	1/15 (6.7)	ND
	SARS-CoV-2	ND	ND	3/54 (5.6)	5/15 (33.3)	ND
	SARS-CoV-2/P	8/26 (11.5)	5/26 (19.2)	14/54 (25.9)	7/15 (46.7)	2/15 (13.3)
v-qPCR/v-RT-qPCR	AdVs	9/12 (75)	7/8 (87.5)	22/23 (95.7)	15/15 (100)	5/6 (83.3)
	HBoV	1/2 (50)	ND	13/16 (81.3)	9/9 (100)	3/3 (100)
	NoV GI	3/4 (75)	ND	12/17 (70.6)	11/12 (91.7)	3/4 (75)
	NoV GII	4/6 (66.7)	ND	28/30 (93.3)	15/15 (100)	6/7 (85.7)
	RoVs	6/9 (66.7)	4/5 (80)	32/34 (94.1)	11/11 (100)	4/5 (80)
	IAV	ND	ND	ND	1/1 (100)	ND
	SARS-CoV-2	ND	ND	1/3 (33.3)	5/5 (100)	0/15 (0)
	SARS-CoV-2/P	3/8 (37.5)	3/5 (60)	9/14 (64.3)	6/7 (85.7)	1/2 (50)

^C^*^)^air samples collected with Coriolis μ impinger, ^M^**^)^air samples collected with MAS-100NT impactor, *AdVs* adenoviruses, *HBoV* human bocavirus, *RoVs* rotaviruses, *NoV GI* Norwalk virus genogroup I, *NoV GII* Norwalk virus genogroup II, *IAV* influenza A virus, *SARS-CoV-2* severe acute respiratory syndrome coronavirus 2, *SARS-CoV-2/P* presumptive SARS-CoV-2 positive/other coronaviruses positive, *ND* not detected.

Taking into account bioaerosol sampling devices, the use of Coriolis μ impinger allowed to detect two types of DNA (AdVs, HBoV) and four types of RNA viruses (NoV GI, NoV GII, RoVs, and presumptive SARS-CoV-2 or other coronaviruses), while MAS-100NT one type of DNA (AdVs) and two types of RNA viruses (RoVs and presumptive SARS-CoV-2 or other coronaviruses). Among the air samples collected with Coriolis μ impinger, 46.2% were AdV positive, 34.6% RoV positive, 23.1% NoV GII positive, 15.4% NoV GI positive, 11.5% presumptive SARS-CoV-2 or other coronaviruses positive, and 7.7% HBoV positive, while in case of MAS-100NT impactor, 30.8% were AdV positive, and equally 19.2% RoV positive and presumptive SARS-CoV-2 or other coronaviruses positive. Viruses were significantly more often detected in the air samples collected with Coriolis μ impinger, than in samples gathered with MAS-100NT impactor (Fisher Exact test: *p* = 0.001).

**Figure 1:**
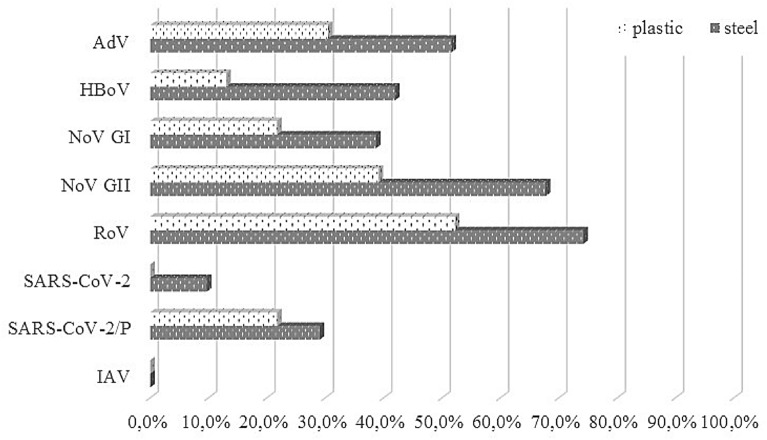
Percentage of positive samples on plastic and steel surfaces. Abbreviations: AdVs – adenoviruses, HBoV – human bocavirus, RoVs – rotaviruses, NoV GI – Norwalk virus genogroup I, NoV GII – Norwalk virus genogroup II, IAV – influenza A virus, SARS-CoV-2 – severe acute respiratory syndrome coronavirus 2, SARS-CoV-2/P – presumptive SARS-CoV-2 positive/other coronaviruses positive.

Laboratory analysis indicated also that 63% of surface swab samples were RoV positive, 55.6% NoV GII positive, 42.6% AdV positive, 31.5% NoV GI positive, 29.6% HBoV positive, 25.9% presumptive SARS-CoV-2 or other coronaviruses positive, and 5.6% SARS-CoV-2 positive. Taking into account the type of surface, positive samples predominated among swabs collected from steel fomites (Fig. 1). However, statistical analyzes showed that, in case of HBoV (Fisher Exact test: *p* = 0.021), NoV GII (Fisher Exact test: *p* = 0.035) and SARS-CoV-2 (Fisher Exact test: *p* = 0.044), the steel surfaces were significantly more often contaminated than plastic ones.

The highest number of positive samples was detected among influent wastewater samples, and was equal to 100% for AdVs and NoV GII, 80% for NoV GI, 73.3% for RoVs, 60% for HBoV, 46.7% for presumptive SARS-CoV-2 or other coronaviruses, 33.3% for SARS-CoV-2, and 6.7% for IAV. In case of effluent wastewater samples, 46.7% were NoV GII positive, 40% AdV positive, 33.3% RoV positive, 26.7% NoV GI positive, 20% HBoV positive, and 13.3% presumptive SARS-CoV-2 or other coronaviruses positive.

Application of v-qPCR/v-RT-qPCR method revealed occurrence of potentially infectious intact viral particles. Percentages of samples containing potentially infectious viruses among all positive samples are presented in Table 2. In case of bioaerosol, the percentage of samples containing potentially infectious viruses among total positive samples ranged from 37.5% (for presumptive SARS-CoV-2 positive/other coronaviruses) to 75% (AdVs, NoV GI) and from 60% (for presumptive SARS-CoV-2 positive/other coronaviruses) to 87.5% (AdVs) for Coriolis μ and MAS-100NT samplers, respectively. Potentially infectious viruses were more often detected in bioaerosol collected with Coriolis μ impinger than with MAS-100NT impactor (Fisher Exact test: *p* = 0.033). In surface swabs, potentially infectious viruses were present from 33.3% (SARS-CoV-2) to 95.7% (AdVs) of positive samples and were more often detected on steel surfaces; however, this difference was not statistically significant (Fisher Exact test: *p* = 0.073). In turn, for influent and effluent wastewaters, the percentage of samples containing potentially infectious viruses among all positive samples ranged from 85.7% (SARS-CoV-2) to 100% (AdVs, HBoV, NoV GII, RoVs, IAV, SARS-CoV-2) and from 50% (presumptive SARS-CoV-2 positive/other coronaviruses) to 100% (HBoV), respectively.

### Quantitative analysis of DNA/RNA viruses in the air samples

The number of viruses detected in the air samples varied between 10^2^–10^4^ gc/m^3^ and between 10^2^ and 10^3^ gc/m^3^ for Coriolis μ and MAS-100NT samplers, respectively (Table 3). In turn, the concentration of potentially infectious viruses revealed by v-qPCR/v-RT-qPCR did not exceed 10^3^ gc/m^3^ for Coriolis μ impinger and 10^2^ gc/m^3^ for MAS-100NT impactor.

**Table 3:** The concentrations of DNA and RNA viruses (gc/m^3^) in air samples collected using Coriolis μ impinger and MAS-100NT impactor.

Viruses		Concentration (gc/m^3^)cx							
		Coriolis μ				MAS-100NT			
		T		P-INF		T		P-INF	
		M	SD	M	SD	M	SD	M	SD
DNA	AdVs	7.03 × 10^3^	3.27 × 10^3^	1.54 × 10^3^	2.51 × 10^3^	4.73 × 10^3^	1.81 × 10^3^	9.48 × 10^2^	1.23 × 10^3^
	HBoV	8.1 × 10^2^	1.69 × 10^2^	4.73 × 10^1^	5.47 × 10^1^	BDL	BDL	BDL	BDL
RNA	NoV GI	1.06 × 10^3^	4.57 × 10^2^	1.05 × 10^2^	8.56 × 10^1^	BDL	BDL	BDL	BDL
	NoV GII	2.22 × 10^3^	1.07 × 10^3^	3.97 × 10^2^	4.61 × 10^2^	BDL	BDL	BDL	BDL
	RoVs	1.52 × 10^4^	2.64 × 10^4^	1.08 × 10^3^	9.01 × 10^2^	3.89 × 10^3^	2.18 × 10^3^	8.74 × 10^2^	4.88 × 10^2^
	IAV	BDL	BDL	BDL	BDL	BDL	BDL	BDL	BDL
	SARS-CoV-2	BDL	BDL	BDL	BDL	BDL	BDL	BDL	BDL
	SARS-CoV-2/P	5.32 × 10^3^	4.56 × 10^3^	2.44 × 10^2^	3.43 × 10^2^	4.5 × 10^2^	4.29 × 10^2^	6.05 × 10^1^	6.69 × 10^1^

*T* total concentration of viruses, *P-INF* concentration of potentially infectious viruses, *M* arithmetic mean value, *SD* standard deviation, *BDL* below detection limit, *AdVs* adenoviruses, *HBoV* human bocavirus, *NoV GI* Norwalk virus genogroup I, *NoV GII* Norwalk virus genogroup II, *RoVs* rotaviruses, *IAV* influenza A virus, *SARS-CoV-2* severe acute respiratory syndrome coronavirus 2, *SARS-CoV-2/P* presumptive SARS-CoV-2 positive/other coronaviruses positive.

Adenoviruses, rotaviruses and presumptive SARS-CoV-2 or other coronaviruses were the only agents detected with both samplers. The concentrations of these viruses were generally higher in samples collected with Coriolis μ impinger, especially in case of AdVs and presumptive SARS-CoV-2 or other coronaviruses (Mann–Whitney tests: *p* = 0.047 and *p* = 0.003, respectively). Regarding potentially infectious viruses, their significantly higher levels were noted for presumptive SARS-CoV-2 or other coronaviruses in Coriolis μ samples (Mann–Whitney test: *p* = 0.004).

All detected potentially infectious viruses were observed within wastewater pumping section and their concentrations there were the highest among examined workplaces (Tables 4). In Coriolis μ and MAS-100NT samples from wastewater pumping area, the average concentrations (and ranges) of AdVs, RoVs as well as presumptive SARS-CoV-2 or other coronaviruses were as follows: 4.41 × 10^3^ gc/m^3^ (1.28–7.41 × 10^3^) and 2.12 × 10^3^ gc/m^3^ (4.41 × 10^2^–2.9 × 10^3^), 1.97 × 10^3^ gc/m^3^ (1.7–2.29 × 10^3^) and 1.2 × 10^3^ gc/m^3^ (1.01–1.4 × 10^3^) as well as 4.87 × 10^2^ gc/m^3^ (4.95–9 × 10^2^) and 1.01 × 10^2^ gc/m^3^ (3.38 × 10^1^–1.64 × 10^2^), respectively. In the air samples collected with Coriolis μ impinger, potentially infectious HBoV, NoV GI and NoV GII were also detected and their highest observed concentrations were equal to 4.73 × 10^1^ gc/m^3^, 2.25 × 10^2^ gc/m^3^ and 7.08 × 10^2^ gc/m^3^, respectively. The presence of potentially infectious viruses was also noted in screens section; however, their average concentrations did not exceed 10^2^ gc/m^3^. Regarding other examined workplaces, none of potentially infectious viruses were detected in air samples from dewatering/thickening sludge section, while in samples collected with Coriolis μ impinger from grit chamber area, the average concentration of potentially infectious AdVs was equal to 8.1 × 10^1^ gc/m^3^.

**Table 4:** The concentrations of DNA and RNA viruses (gc/m^3^) in air samples collected using Coriolis μ impinger and MAS-100NT impactor at examined WWTP sampling sites.

Viruses			Sample		Concentration (gc/m^3^)			
					1	2	3	4
Coriolis®μ impinger	DNA	AdV	T	M	1.06 × 10^4^	7.07 × 10^3^	2.48 × 10^3^	3.15 × 10^3^
				SD	1.49 × 10^3^	1.6 × 10^3^	1.7 × 10^1^	6.03 × 10^2^
			P-INF	M	4.41 × 10^3^	1.99 × 10^2^	8.1 × 10^1^	BDL
				SD	2.55 × 10^3^	1.23 × 10^2^	1.27 × 10^1^	–
		HBoV	T	M	8.1 × 10^2^	BDL	BDL	BDL
				SD	1.69 × 10^2^	–	–	–
			P-INF	M	4.73 × 10^1^	BDL	BDL	BDL
				SD	5.47 × 10^1^	–	–	–
	RNA	NoV GI	T	M	1.35 × 10^3^	1.24 × 10^3^	BDL	4.01 × 10^2^
				SD	6.36 × 10^0^	3.15 × 10^2^	–	6.36 × 10^0^
			P-INF	M	2.25 × 10^2^	9.68 × 10^1^	BDL	BDL
				SD	1.27 × 10^1^	4.5 × 10^0^	–	–
		NoV GII	T	M	3.14 × 10^3^	1.5 × 10^3^	BDL	8.96 × 10^2^
				SD	6.05 × 10^2^	2.53 × 10^2^	–	6.36 × 10^0^
			P-INF	M	7.08 × 10^2^	2.39 × 10^2^	BDL	BDL
				SD	4.79 × 10^2^	1.16 × 10^1^	–	–
		RoV	T	M	3.24 × 10^4^	1.71 × 10^3^	BDL	9.27 × 10^2^
				SD	3.29 × 10^3^	3.81 × 10^2^	–	3.82 × 10^1^
			P-INF	M	1.97 × 10^3^	3.06 × 10^2^	BDL	BDL
				SD	2.36 × 10^2^	4.74 × 10^2^	–	–
		IAV	T	M	BDL	BDL	BDL	BDL
				SD	–	–	–	–
			P-INF	M	BDL	BDL	BDL	BDL
				SD	–	–	–	–
		SARS-CoV-2	T	M	BDL	BDL	BDL	BDL
				SD	–	–	–	–
			P-INF	M	BDL	BDL	BDL	BDL
				SD	–	–	–	–
		SARS-CoV-2/P	T	M	6.84 × 10^3^	3.79 × 10^3^	BDL	BDL
				SD	1.9 × 10^3^	5.97 × 10^3^	–	–
			P-INF	M	4.87 × 10^2^	BDL	BDL	BDL
				SD	3.42 × 10^2^	–	–	–
MAS-100NT impactor	DNA	AdV	T	M	6.82 × 10^3^	3.44 × 10^3^	3.6 × 10^3^	BDL
				SD	8.2 × 10^2^	7.15 × 10^2^	6.36 × 10^0^	–
			P-INF	M	2.12 × 10^3^	3.06 × 10^2^	BDL	BDL
				SD	1.33 × 10^3^	2.26 × 10^2^	–	–
		HBoV	T	M	BDL	BDL	BDL	BDL
				SD	–	–	–	–
			P-INF	M	BDL	BDL	BDL	BDL
				SD	–	–	–	–
	RNA	NoV GI	T	M	BDL	BDL	BDL	BDL
				SD	–	–	–	–
			P-INF	M	BDL	BDL	BDL	BDL
				SD	–	–	–	–
		NoV GII	T	M	BDL	BDL	BDL	BDL
				SD	–	–	–	–
			P-INF	M	BDL	BDL	BDL	BDL
				SD	–	–	–	–
		RoV	T	M	6.04 × 10^3^	1.5 × 10^3^	BDL	4.73 × 10^3^
				SD	5.12 × 10^2^	2.13 × 10^2^	–	2.55 × 10^1^
			P-INF	M	1.2 × 10^3^	9.86 × 10^2^	BDL	BDL
				SD	2.11 × 10^2^	5.42 × 10^1^	–	–
		IAV	T	M	BDL	BDL	BDL	BDL
				SD	–	–	–	–
			P-INF	M	BDL	BDL	BDL	BDL
				SD	–	–	–	–
		SARS-CoV-2	T	M	BDL	BDL	BDL	BDL
				SD	–	–	–	–
			P-INF	M	BDL	BDL	BDL	BDL
				SD	–	–	–	–
		SARS-CoV-2/P	T	M	7.32 × 10^2^	2.64 × 10^1^	BDL	BDL
				SD	3.03 × 10^2^	3.84 × 10^0^	–	–
			P-INF	M	1.01 × 10^2^	BDL	BDL	BDL
				SD	5.62 × 10^1^	–	–	–

Notes: 1 – wastewater pumping section, 2 – screens section, 3 – grit chamber section, 4 – dewatering/thickening sludge section, AdVs – adenoviruses, HBoV – human bocavirus, NoV GI – Norwalk virus genogroup I, NoV GII – Norwalk virus genogroup II, RoVs – rotaviruses, IAV – influenza A virus, SARS-CoV-2 – severe acute respiratory syndrome coronavirus 2, SARS-CoV-2/P – presumptive SARS-CoV-2 positive/other coronaviruses positive, T – total concentration of viruses, P-INF – concentration of potentially infectious viruses, M – arithmetic mean value, SD – standard deviation, BDL – below detection limit.

### Influence of temperature and relative humidity of the air on concentration of viruses

For the examined sampling sites, the air temperature ranged from 13.9 °C in wastewater pumping section to 26.4 °C in dewatering/thickening sludge section. The highest relative humidity (RH) of the air was observed in wastewater pumping Sect. (64.3%), while the lowest within grit chamber Sect. (32.9%) (Table 5). Statistical analysis showed that temperature negatively influenced the concentrations of all tested potentially infectious airborne viruses (Spearman correlation – in all cases: R = –0.536 to –0.951 at *p* < 0.05). The strongest negative correlations were observed for RoVs (R = –0.951 at *p* = 0.000) and AdVs (R = –0.924 at *p* = 0.000). Contrary to temperature, the RH positively correlated with the concentrations of all tested potentially infectious airborne viruses (in all cases: R = 0.710 to 0.747 at *p* < 0.05).

**Table 5:** Temperature and relative humidity of the air at examined WWTP sampling sites.

WWTP sites	Temperature		Relative humidity	
	M	SD	M	SD
1	13.9	0.9	64.3	3.7
2	21.2	2.4	36.3	4.9
3	25.3	0.6	32.9	1.4
4	26.4	0.6	46.4	15.2

*M* arithmetic mean value, *SD* standard deviation, *1* wastewater pumping section, *2* screens section, *3* grit chamber section, *4* dewatering/thickening sludge section.

### Quantitative analysis of DNA/RNA viruses in surface swab samples

The number of viruses detected in surface swab samples varied between 10^3^–10^6^ gc/100 cm^2^. The use of v-qPCR/v-RT-qPCR revealed that the concentration of potentially infectious intact viral particles on surfaces vary from 10^1^ gc/100 cm^2^ to 10^4^ gc/100 cm^2^ (Table 6). The highest concentrations of potentially infectious viruses were detected on steel surfaces for RoVs with the mean value of 5.37 × 10^4^ gc/100 cm^2^ (range 1.21 × 10^3^–2.04 × 10^4^) and on plastic surfaces for NoV GII with mean value of 1.41 × 10^4^ gc/100 cm^2^ (range 2.04 × 10^2^–1.09 × 10^5^). The average concentrations of potentially infectious AdVs, HBoV, NoV GI, and NoV GII on steel surfaces did not exceed 10^4^ gc/100 cm^2^, while in the cases of SARS-CoV-2 and presumptive SARS-CoV-2 or other coronaviruses, their concentrations were below 10^3^ gc/100 cm^2^. Also the average concentrations of potentially infectious HBoV, NoV GI, and RoVs on plastic surfaces did not exceed 10^4^ gc/100 cm^2^, while in the case of AdVs and presumptive SARS-CoV-2 or other coronaviruses their levels were less than 10^3^ gc/100 cm^2^. The comparison of potentially infectious virus concentration on steel and plastic surfaces revealed significantly higher contamination only in the case of plastic surfaces contaminated with potentially infectious HBoV (Mann–Whitney test: *p* = 0.006).

**Table 6:** The concentrations of DNA and RNA viruses (gc/100 cm^2^) in positive swab samples from steel and plastic surfaces.

Viruses		Concentration (gc/100 cm^2^)							
		ST				P			
		T		P-INF		T		P-INF	
		M	SD	M	SD	M	SD	M	SD
DNA	AdVs	1.5 × 10^4^	1.28 × 10^4^	2.22 × 10^3^	2.3 × 10^3^	4.7 × 10^3^	6.19 × 10^3^	6.68 × 10^2^	7.66 × 10^2^
	HBoV	5.47 × 10^3^	7.45 × 10^3^	2.21 × 10^3^	4.73 × 10^3^	2.61 × 10^4^	2.87 × 10^4^	9.22 × 10^3^	1 × 10^4^
RNA	NoV GI	2.59 × 10^5^	5.36 × 10^5^	5.45 × 10^3^	1.32 × 10^4^	1.99 × 10^5^	2.87 × 10^5^	1.5 × 10^3^	2.34 × 10^3^
	NoV GII	5.98 × 10^4^	1.34 × 10^5^	7.35 × 10^3^	1.78 × 10^4^	2.25 × 10^4^	3.44 × 10^4^	1.41 × 10^4^	3.74 × 10^4^
	RoVs	2.76 × 10^6^	5.41 × 10^6^	5.37 × 10^4^	2.2 × 10^5^	1.58 × 10^6^	3.07 × 10^6^	6.9 × 10^3^	3.47 × 10^4^
	IAV	BDL	BDL	BDL	BDL	BDL	BDL	BDL	BDL
	SARS-CoV-2	7.17 × 10^3^	5.37 × 10^3^	5.8 × 10^1^	8.99 × 10^1^	BDL	BDL	BDL	BDL
	SARS-CoV-2/P	6.6 × 10^4^	1.52 × 10^5^	1.37 × 10^2^	1.17 × 10^2^	1.16 × 10^4^	1.31 × 10^4^	2.7 × 10^2^	1.94 × 10^2^

*ST* steel, *P* plastic, *T* total concentration of viruses, *P-INF* concentration of potentially infectious viruses, *M* arithmetic mean value, *SD* standard deviation, *BDL* below detection limit, *AdVs* adenoviruses, *HBoV* human bocavirus, *NoV GI* Norwalk virus genogroup I, *NoV GII* Norwalk virus genogroup II, *RoVs* rotaviruses, *IAV* influenza A virus, *SARS-CoV-2* severe acute respiratory syndrome coronavirus 2, *SARS-CoV-2/P* presumptive SARS-CoV-2 positive/other coronaviruses positive.

Taking into account the metal surfaces, the significant differences in concentrations of potentially infectious virions depending of sampling site were observed for AdV, HBoV, RoV and NoV GII viruses (Kruskal–Wallis tests: *p* = 0.03, *p* = 0.012, *p* = 0.001, and *p* = 0.014, respectively), while in case of plastic surfaces for AdV, NoV GII and presumptive SARS-CoV-2 viruses (Kruskal–Wallis tests: *p* = 0.036, *p* = 0.002, and *p* = 0.01, respectively). The sampling site dependent differences in concentrations of potentially infectious NoV GI virions were not significant for both steel and plastic surfaces (Table 7).

**Table 7:** The concentrations of DNA and RNA viruses (gc/100 cm^2^) in positive swab samples from steel and plastic surfaces at examined WWTP sampling sites.

Viruses		Concentration (gc/100 cm^2^)									
		Sample		1		2		3		4	
				ST	P	ST	P	ST	P	ST	P
DNA	AdVs	T	M	2.87 × 10^3^	9.26 × 10^3^	1.66 × 10^4^	4.68 × 10^2^	1.2 × 10^4^	6.26 × 10^3^	1.83 × 10^4^	BDL
			SD	2.76 × 10^3^	1.71 × 10^3^	1.54 × 10^4^	4.79 × 10^2^	2.55 × 10^1^	1.91 × 10^3^	2.21 × 10^3^	–
		P-INF	M	1.44 × 10^2^	6.15 × 10^2^	2.63 × 10^3^	1.7 × 10^2^	2.7 × 10^2^	3.8 × 10^2^	2.73 × 10^3^	BDL
			SD	2.4 × 10^1^	9.25 × 10^1^	2.49 × 10^3^	5.6 × 10^1^	8.49 × 10^0^	4.3 × 10^2^	1.85 × 10^3^	–
	HBoV	T	M	2.67 × 10^3^	1.43 × 10^4^	1.25 × 10^4^	6.23 × 10^4^	1.55 × 10^3^	BDL	2.12 × 10^3^	1.63 × 10^3^
			SD	2.08 × 10^3^	5.09 × 10^1^	1.05 × 10^4^	1.4 × 10^2^	1.19 × 10^2^	–	1.09 × 10^3^	1.53 × 10^2^
		P-INF	M	1.08 × 10^3^	2.34 × 10^2^	5.68 × 10^3^	2.21 × 10^4^	6.35 × 10^2^	BDL	1.41 × 10^2^	1.38 × 10^3^
			SD	9.59 × 10^2^	7.64 × 10^1^	7.65 × 10^3^	3.56 × 10^2^	7.34 × 10^2^	–	1.14 × 10^2^	8.91 × 10^1^
RNA	NoV GI	T	M	7.4 × 10^3^	BDL	4.28 × 10^5^	1.44 × 10^5^	8.94 × 10^2^	BDL	4.65 × 10^4^	2.36 × 10^5^
			SD	2.78 × 10^3^	–	6.59 × 10^5^	1.66 × 10^5^	4.24 × 10^1^	–	6.12 × 10^3^	3.58 × 10^5^
		P-INF	M	6.06 × 10^2^	BDL	8.76 × 10^3^	3.21 × 10^2^	BDL	BDL	1.44 × 10^3^	1.72 × 10^3^
			SD	3.68 × 10^2^	–	1.67 × 10^4^	3.71 × 10^2^	–	–	1.22 × 10^3^	2.6 × 10^3^
	NoV GII	T	M	1.05 × 10^4^	BDL	1.32 × 10^5^	5.99 × 10^4^	1.48 × 10^3^	1.09 × 10^3^	3.38 × 10^3^	5.04 × 10^3^
			SD	9.95 × 10^3^	–	1.84 × 10^5^	3.82 × 10^4^	7.84 × 10^2^	6.93 × 10^2^	4.2 × 10^3^	3.55 × 10^3^
		P-INF	M	5.36 × 10^3^	BDL	1.38 × 10^4^	4.12 × 10^4^	3.44 × 10^2^	5.04 × 10^2^	8.42 × 10^2^	4.92 × 10^2^
			SD	5.05 × 10^3^	–	2.69 × 10^4^	5.27 × 10^4^	4.14 × 10^2^	1.96 × 10^1^	1.24 × 10^3^	4.22 × 10^2^
	RoVs	T	M	7.11 × 10^5^	3.7 × 10^6^	5.62 × 10^6^	1.69 × 10^6^	1.46 × 10^6^	1.21 × 10^5^	6.6 × 10^3^	6.01 × 10^5^
			SD	5.18 × 10^5^	4.27 × 10^6^	7.33 × 10^6^	3.25 × 10^6^	2.4 × 10^4^	3.87 × 10^2^	2.11 × 10^3^	5.38 × 10^5^
		P-INF	M	1.03 × 10^4^	8.38 × 10^3^	1.14 × 10^5^	7.07 × 10^3^	1.48 × 10^4^	2.11 × 10^3^	8.64 × 10^2^	9.72 × 10^3^
			SD	8.65 × 10^3^	7.59 × 10^3^	2.91 × 10^5^	5.9 × 10^3^	3.46 × 10^3^	1.48 × 10^2^	7.57 × 10^2^	5.96 × 10^3^
	IAV	T	M	BDL	BDL	BDL	BDL	BDL	BDL	BDL	BDL
			SD	–	–	–	–	–	–	–	–
		P-INF	M	BDL	BDL	BDL	BDL	BDL	BDL	BDL	BDL
			SD	–	–	–	–	–	–	–	–
	SARS-CoV-2	T	M	2.4 × 10^2^	BDL	1.06 × 10^4^	BDL	BDL	BDL	BDL	BDL
			SD	1.7 × 10^1^	–	1.34 × 10^2^	–	–	–	–	–
		P-INF	M	1.74 × 10^2^	BDL	BDL	BDL	BDL	BDL	BDL	BDL
			SD	8.49 × 10^0^	–	–	–	–	–	–	–
	SARS-CoV-2/P	T	M	1.7 × 10^3^	2.62 × 10^4^	9.88 × 10^4^	2.09 × 10^3^	2.7 × 10^2^	BDL	BDL	BDL
			SD	1.78 × 10^3^	5.2 × 10^3^	1.81 × 10^5^	2.44 × 10^3^	2.55 × 10^1^	–	–	–
		P-INF	M	3.24 × 10^2^	4.74 × 10^2^	9.9 × 10^1^	1.61 × 10^2^	BDL	BDL	BDL	BDL
			SD	1.08 × 10^2^	1.55 × 10^1^	1.07 × 10^1^	9.35 × 10^1^	–	–	–	–

*1* wastewater pumping section, *2* screens section, *3* grit chamber section, *4* dewatering/thickening sludge section, *ST* steel, *P* plastic, *T* total concentration of viruses, *P-INF* concentration of potentially infectious viruses, *M* arithmetic mean value, *SD* standard deviation, *BDL* below detection limit, *AdVs* adenoviruses, *HBoV* human bocavirus, *NoV GI* Norwalk virus genogroup I, *NoV GII* Norwalk virus genogroup II, *RoVs* rotaviruses, *IAV* influenza A virus, *SARS-CoV-2* severe acute respiratory syndrome coronavirus 2, *SARS-CoV-2/P* presumptive SARS-CoV-2 positive/other coronaviruses positive.

The highest concentrations of potentially infectious AdVs were detected on steel surfaces in dewatering/thickening sludge (range 8.28 × 10^2^–4.67 × 10^3^ gc/100 cm^2^) and screens (range 1.68 × 10^2^–7.21 × 10^3^ gc/100 cm^2^) sections, whereas NoV GI and RoVs on steel surfaces in screens section (ranges 2.52 × 10^2^–4.77 × 10^4^ gc/100 cm^2^ and 3.53 × 10^3^–1.07 × 10^6^ gc/100 cm^2^, respectively). Additionally, the steel surfaces in wastewater pumping section were the only ones where potentially infectious SARS-CoV-2 was detected (range 1.68 × 10^2^–1.8 × 10^2^ gc/100 cm^2^). In turn, the highest concentrations of potentially infectious HBoV and NoV GII viruses were observed on plastic surfaces in screens section (ranges 2.18 × 10^4^–2.23 × 10^4^ gc/100 cm^2^ and 3.53 × 10^3^–1.09 × 10^5^ gc/100 cm^2^, respectively), while presumptive SARS-CoV-2 or other coronaviruses on plastic surfaces in wastewater pumping section (range 1.44 × 10^2^–2.52 × 10^2^ gc/100 cm^2^).

### Quantitative analysis of DNA/RNA viruses in influent and effluent wastewater samples

The number of viruses in influent and effluent wastewater samples ranged between 10^4^ and 10^7^ gc/L and between 10^2^ and 10^4^ gc/L, respectively (Table 8). The highest total concentration of viruses was observed in the cases of AdVs with the mean value of 9.84 × 10^7^ gc/L (range 3.6 × 10^3^–8.45 × 10^8^) and RoVs with the mean value of 1.14 × 10^7^ gc/L (range 2.27 × 10^5^–7.68 × 10^7^) in influent and in the case of NoV GI with the mean value of 9.29 × 10^4^ gc/L (range 7.74 × 10^3^–1.42 × 10^5^) in effluent samples.

**Table 8:** The concentrations of DNA and RNA viruses (gc/L) in positive influent and effluent wastewater samples.

Viruses		Concentration (gc/L)			
				Influent samples	Effluent samples
DNA	AdV	T	M	9.84 × 10^7^	7.24 × 10^3^
			SD	2.16 × 10^8^	3.31 × 10^3^
		P–INF	M	1.6 × 10^7^	3.83 × 10^3^
			SD	3.45 × 10^7^	3.48 × 10^3^
	HBoV	T	M	2.02 × 10^5^	6.32 × 10^4^
			SD	5.09 × 10^5^	6.39 × 10^4^
		P–INF	M	6.84 × 10^4^	5 × 10^4^
			SD	5.19 × 10^5^	5.64 × 10^4^
RNA	NoV GI	T	M	6.15 × 10^6^	9.29 × 10^4^
			SD	2.01 × 10^7^	5.68 × 10^4^
		P-INF	M	2.09 × 10^6^	3.06 × 10^4^
			SD	6.79 × 10^6^	3.97 × 10^4^
	NoV GII	T	M	2.1 × 10^6^	1.8 × 10^4^
			SD	6.85 × 10^6^	9.02 × 10^3^
		P-INF	M	4.2 × 10^5^	5.85 × 10^3^
			SD	8.88 × 10^5^	5.63 × 10^3^
	RoV	T	M	1.14 × 10^7^	2.38 × 10^4^
			SD	1.94 × 10^7^	4.18 × 10^4^
		P-INF	M	1.04 × 10^6^	4.27 × 10^3^
			SD	1.2 × 10^6^	3.94 × 10^3^
	IAV	T	M	1.31 × 10^5^	BDL
			SD	4.84 × 10^3^	–
		P-INF	M	1.23 × 10^5^	BDL
			SD	2.55 × 10^3^	–
	SARS-CoV-2	T	M	3.46 × 10^4^	BDL
			SD	1.98 × 10^4^	–
		P-INF	M	9.63 × 10^3^	BDL
			SD	4.4 × 10^3^	–
	SARS-CoV-2/P	T	M	1.64 × 10^5^	5.97 × 10^3^
			SD	8.59 × 10^4^	6.68 × 10^3^
		P-INF	M	1.19 × 10^5^	7.11 × 10^2^
			SD	7.34 × 10^4^	8.23 × 10^2^

*T* total concentration of viruses, *P-INF* concentration of potentially infectious viruses, *M* arithmetic mean value, *SD* standard deviation, *BDL* below detection limit, *AdVs* adenoviruses, *HBoV* human bocavirus, *NoV GI* Norwalk virus genogroup I, *NoV GII* Norwalk virus genogroup II, *RoVs* rotaviruses, *IAV* influenza A virus, *SARS-CoV-2* severe acute respiratory syndrome coronavirus 2, *SARS-CoV-2/P* presumptive SARS-CoV-2 positive/other coronaviruses positive.

The application of v-qPCR/v-RT-qPCR revealed that the concentrations of potentially infectious intact viral particles in influent samples ranged between 10^3^–10^7^ gc/L, while in effluent samples did not exceed 10^4^ gc/L. The highest levels of potentially infectious viruses in influent samples were observed for AdVs (range 3.56 × 10^4^–1.28 × 10^8^ gc/L), NoV GI (range 6.84 × 10^3^–2.42 × 10^7^ gc/L), and RoVs (range 1.17 × 10^5^–3.82 × 10^6^ gc/L). In turn, in effluent samples, the highest concentrations of potentially infectious viruses were observed for HBoV (range 6.12 × 10^3^–1.21 × 10^6^ gc/L) and NoV GI (range 5.22 × 10^3^–9.38 × 10^4^ gc/L).

## Discussion

This study revealed that both gastrointestinal and respiratory viruses were present at workplaces in WWTPs. Their detection in influent samples indicates their wastewater-borne origin. The presence of viruses in occupational environments has been proved in several studies using PCR-based methods^1,36,37,39,49,50^; however, it should be clearly pointed out that RT-qPCR/qPCR enables both qualitative and quantitative analyzes of viral RNA/DNA, but does not allow to assess viral infectious ability^51^. To the best of our knowledge, this is the first investigation qualitatively and quantitatively analyzing the presence of the most common gastrointestinal and respiratory viruses at workplaces in WWTPs and through the coupling of PMA dye with qPCR/RT-qPCR assays discriminating the potentially infectious and disintegrated viral particles in airborne, surface, and waterborne samples.

This study showed that both above mentioned groups of viruses were dispersed in the air at workplaces in WWTPs. Viruses, including potentially infectious ones, were significantly more often detected in the air samples collected with Coriolis μ impinger, than with MAS-100NT impactor. Coriolis μ sampler collected particles into a liquid medium, while MAS-100NT device utilized bi-phase medium consisting of solid agar covered with a thin layer of liquid viral transport medium. Both samplers, however, have limitations when considering their use and induce particle loss. For single-stage impactors, bouncing of particles (when they strike the impaction surface) can lead to undersampling, destruction of collected particles, and decrease of collection efficiency. In turn, the cyclonic samplers were so far successfully used in several studies for collection of airborne viral particles^38,52–55^. Impingers are not sensitive to overloading or undersampling as they provide generally ‘gentle’ particle collection. However, evaporation of the sampling liquid and reaerosolization of already trapped particles may bias the sampling results. As it was shown in this study, the strike of viral particles against the agar surface (even if it is covered with thin liquid layer) seems to be an important factor destroying viral particles in single-stage impactor. Moreover, the different sizes and airborne behaviors of tested viruses may also influence the capture efficiency of both samplers. Viruses can occur in airborne state in varied forms: as droplets that are relatively large and largely liquid (> 20 μm) or as medium (5–20 μm) and small (≤ 5 μm) size particles that may be composed of either liquid or solid materials. Fine particles can remain airborne for extended periods of time, especially if they are mostly composed of water (as the water evaporates, the viral particles become smaller in size over time)^56^. It is proved that single virus particles may exist in the air, but they tend to aggregate rapidly and/or may be ‘protected’ by larger particles, being adsorbed on their surfaces^40,57^. Viruses aggregated to larger particles show higher survivability compared to particles, which real dimensions are close to the actual size of the virions^58,59^. Some authors indicated that the best isolation efficiency of viable intact viruses was observed in case of aggregated particles larger than 2.1 µm^58,60^. In case of NoVs and RoVs, their highest environmental concentrations in airborne state were noted for particles with diameters of > 4.5 µm and 9 µm, respectively^38,41^. As the performed study showed the bioaerosol sampling utilizing cyclone with liquid collection medium seems to be a method of choice due to its fast collection of large air volume providing a high recovery rate for viral particles with broad spectrum of sizes. These features have high practical value, especially in occupational environments like WWTPs, where humidity conditions are very variable and other sampling methods (such as e.g. filtration or impaction) are not advisable^40,41^. At workplaces in the studied WWTPs, the most frequently isolated viruses belonged to AdV, RoV, and NoV groups, reaching up to 10^3^ gc/m^3^, 10^4^ gc/m^3^ and 10^3^ gc/m^3^ of potentially infectious intact particles, respectively. It can be explained with the fact that non-enveloped viruses tend to be more stable in high RH conditions and, even being airborne, they could remain infectious for a longer period of time^1^. As it was showed the highest concentrations of airborne viruses were detected in wastewater pumping section (up to 10^4^ gc/m^3^), i.e. in the location where the lowest temperature and the highest RH were observed. For virus-containing aerosols, both these microclimate parameters have the key influence (i.e. temperature negative, while RH positive) on their survival in the air^56^. In turn, potentially infectious presumptive SARS-CoV-2 or other coronaviruses, which represent enveloped viruses, were present in the air of wastewater pumping section only, reaching concentrations at the level of 10^2^ gc/m^3^. The occurrence of those viruses within described area results probably from aerosolization of raw sewage during pumping and was stabilized by high RH (64.3%) and low temperature (13.9 °C). Such picture is consistent with observations by Morris et al.^61^, who found that coronaviruses, including SARS-CoV-2, survive better in low temperature (about 10 °C) and at high RH (over 60%).

Viruses from AdV, RoV, and NoV groups were detected in WWTPs by different research teams with the concentrations reaching 10^6^ gc/m^3^ for AdVs, 10^7^ gc/m^3^ for RoVs, 10^3^ gc/m^3^ for NoV GI, and 10^2^ gc/m^3^ for NoV GII^1,38,62^. The results obtained in this study indicated that the total virus and potentially infectious virion concentrations of AdVs and RoVs were lower and did not exceed 10^4^ and 10^3^ gc/m^3^, respectively; however, for both NoV genotypes were on the same levels. There is lack of information regarding the concentrations of coronaviruses in the air of WWTPs; however, airborne transmission of SARS viruses with droplets containing wastewater is very probable^63,64^. This study revealed that the concentrations of potentially infectious presumptive SARS-CoV-2 or other coronaviruses reached the level of 9 × 10^2^ gc/m^3^ in wastewater pumping section; however, no cases of COVID-19 among WWTP workers were observed. Hence, either the identified viruses were animal coronaviruses not harmful for humans or detected particles, even though they were intact, they lost infectious abilities.

In this study, gastrointestinal and respiratory viruses were also detected on surfaces at workplaces in WWTPs, reaching in case of potentially infectious intact viral particles the concentrations of 10^5^ gc/100 cm^2^. Many studies have documented the possibility of virus transfer from hands to the surfaces of touched objects and back, which may play an important role in spreading of viral infections^42,65–67^. The persistence and stability of viruses vary and depend on many biological (e.g. the type of virus, the presence of microorganisms that can show protective effects against drying and disinfectants) and environmental (e.g. temperature, RH, sunlight exposure, composition of colonized medium) factors^56,68^.

Viruses may remain infectious for extended period of time after deposition on objects. For example, the persistence of clinically relevant viruses on dry inanimate surfaces may range from 3 to 96 h for SARS associated virus and other coronaviruses, from 8 h to 7 days for NoVs, from 1 to 2 days for influenza virus, from 6 days to 2 months for RoVs, and from 7 days to 3 months for AdVs^69,70^.

According to Abad et al.^71^, viruses usually survive longer on non-porous surfaces compared to porous ones. In the present study, the swab samples from non-porous steel and plastic surfaces were analyzed.

It was found that viral particles, especially HBoV, NoV GII and SARS-CoV-2, were more often detected on steel surfaces than on plastic ones. This observation can be explained by the prolonged persistence of viruses on steel and other metal surfaces, which can be up to 120 days^72^. However, taking into account potentially infectious viral particle concentrations, it was found that both these inanimate surfaces at workplaces in WWTPs were contaminated to the same degree. The only exception in this case was noticed for HBoV. Its significantly higher concentrations on plastic than on steel surfaces could result from both the extreme stability of this pathogen and its high resistance to disinfectants^72^. The only exception was noticed for HBoV. Its significantly higher concentrations on plastic than on steel surfaces could be explained by both the extreme stability of this pathogen and its high resistance to disinfectants^73^.

The potentially infectious intact viral particles were frequently detected among virus-positive surface swab samples with the highest (above 90%) prevalence of AdVs, RoVs, and NoV GII. The DNA viruses, like AdVs, are usually more resistant to degradation than RNA viruses; however, RoVs and NoVs has been shown to tolerate a wide range of harsh environmental conditions like the presence of free chlorine, chemical disinfectants, extreme temperatures and humidities^74,75^. Thus, special measures should be applied to all surfaces and equipment having a direct contact with wastewater to remove viral contamination (e.g. the use of proper disinfectants degrading both RNA and DNA viruses). As some authors indicate that there are no direct relationships between sensitivity to UV light and the virion size, type of nucleic acid or presence/absence of the envelope, the diverse resistance of viruses to UV radiation should be taken into account, when UV light is intended to be used for inactivation of infectious viruses in WWTP environment^76^.

The abundance and diversity of pathogenic viruses, including potentially infectious intact viral particles in influent and effluent wastewater, result in their prevalence at workplaces in WWTPs. The concentrations of potentially infectious viral particles in influent wastewater reached up to 10^7^ gc/L for AdVs, 10^6^ gc/L for NoV GII and RoVs, 10^5^ gc/L for HBoV, IAV, and presumptive SARS-CoV-2 or other coronaviruses, and 10^3^ gc/L for SARS-CoV-2. Potentially infectious viruses were also detected in effluent samples in concentrations ranged from 10^2^ gc/L for presumptive SARS-CoV-2 or other coronaviruses to 10^4^ gc/L for HBoV and NoV GI. The obtained results were consistent with the data gathered by Corpuz et al.^36^. The presence of viruses in wastewater, and thus at workplaces of WWTPs, is closely related to the prevalence of these pathogens in the population. In this study, IAV was not detected in the majority of influent samples, suggesting that the community of the area served by the selected WWTPs was free of this pathogen. An incidentally detected positive sample among analyzed influents suggests that this sample may have been contaminated with bird feces, which are the natural reservoirs of IAV. The lack of IAV nucleic acids (noted in WWTPs and effluent samples) also suggests that IAV is susceptible to environmental degradation^26^.

The hitherto performed evaluations show that the application of PCR-based methods allows to detect both infectious and disintegrated non-infectious viral particles. Although coupling of PMA with qPCR and RT-qPCR has been successfully applied to distinguish infectious and inactivated viral particles in river water, raw manure, soil and food samples, there is lack of information about possible application of these methods in work environment research^31,34,35,48,76,77^. The results of this study showed that the application of v-qPCR/v-RT-qPCR allowed to discriminate potentially infectious intact viral particles and disintegrated virions in the air, surface, and wastewater samples. The ‘classic’ PCR methods are not able to discriminate between potentially infectious and non-infectious viral particles, which can lead to an overestimation of the target viruses. Hence, the positive results obtained in this way should be taken with precautions. Potentially infectious viral particle concentrations detected with v-qPCR/v-RT-qPCR in this study were usually lower (about one to three orders of magnitude) than the concentrations of total viral particles detected with q-PCR/RT-qPCR methods. The latter mentioned methods give more reliable information regarding actual contamination of sampling sites with potentially harmful viral particles. The application of v-qPCR/v-RT-qPCR allows eliminating the number of damaged viral particles from the results and providing information about potentially infectious intact viruses only. On the other hand, not all intact viral particles remain infectious in the environment. Hence, the concentrations of potentially infectious viruses detected with v-qPCR/v-RT-qPCR may be overestimated and further in vitro investigations are needed to define their real infectivity and subsequent real influence on workers’ health. Moreover, as some authors point to biological activity of non-infectious viral particles, which may induce response of host cells, their adverse role for human health should not be neglected^78^.

This study confirmed that WWTP workers were exposed to airborne viral particles, to viruses deposited on surfaces as well as present in influent and effluent wastewater samples. Such massive exposure may lead to the appearance of different infections^79^. Numerous studies have reported that gastrointestinal and respiratory symptoms (e.g. nausea, vomiting, cough, diarrhea, and fever) were observed more frequently among WWTP workers than in general population and may result from exposure to viruses during occupational activities^1,80^. Some viruses (like AdVs) cause generally mild respiratory tract infections, which are self-limiting and generally asymptomatic despite the virologic and serologic proof of infection^81^.

For SARS-CoV-2 viruses, there is evidence (albeit limited) that the minimum infectious dose in humans is greater than 100 particles^82^. In turn, inhalation of infectious dose of RoVs (which is below 100 viral particles) or NoVs (which is about 10 viral particles) may end up in adverse health outcomes^38,83^. According to Musher^82^, inhalation of 5 human adenovirus particles may cause disease in susceptible individuals and even the possibility of infections due to the inhalation of gastrointestinal viruses (with subsequent deglutition of virions deposited within oral cavity) cannot be also excluded. Although, it is difficult to directly compare the infectious doses expressed in viral genome copies with those given in the number of viral particles as these two measures are not equivalent to each other, the researchers have been constantly looking for such links to facilitate the exposure assessment to viruses and evaluate their influence on human health^84–86^.

## Conclusions

Both gastrointestinal and respiratory viruses were present in the air and on surfaces at workplaces as well as in influent and effluent samples from WWTPs and as such may pose an occupational risk for workers. The most frequently isolated viruses, with the highest concentrations reaching up to 10^3^ gc/m^3^ and up to 10^4^ gc/100 cm^2^ of potentially infectious intact virions, were AdVs, RoVs, and NoVs. In the same time, potentially infectious viral particles of SARS-CoV-2 and presumptive SARS-CoV-2 or other coronaviruses were detected in concentrations up to 10^2^ gc/m^3^ and 10^2^ gc/100 cm^2^. Although, the most contaminated area was in general the wastewater pumping section, the potentially infectious viruses occurred within all workplaces involved in wastewater treatment processes. Hence, the risk of infection increases especially in situations where personal hygiene is inadequate (e.g. hand washing is not proper and not enough frequent, there is a lack of personal preventive measure within the areas where bioaerosol forming process are present, eating or drinking at the workplace etc.). To reduce the probability of virus transmission, efficient cleaning procedures degrading viral particles for frequently touched surfaces and objects should be introduced and the use of personal protective equipment, especially within areas where bioaerosol particles are aerosolized, should be mandatory. In this context, both identification and quantification of potentially infectious viruses in WWTPs and other occupational environments with high abundance of microbial contaminants are an important part of safety work management and proper health risk assessment. The application of v-qPCR/v-RT-qPCR represents a big step forward in analysis of viruses in different environmental matrices allowing better interpreting the workplace exposure to these emerging pollutants and should be included in the monitoring procedures for occupational biohazards.
